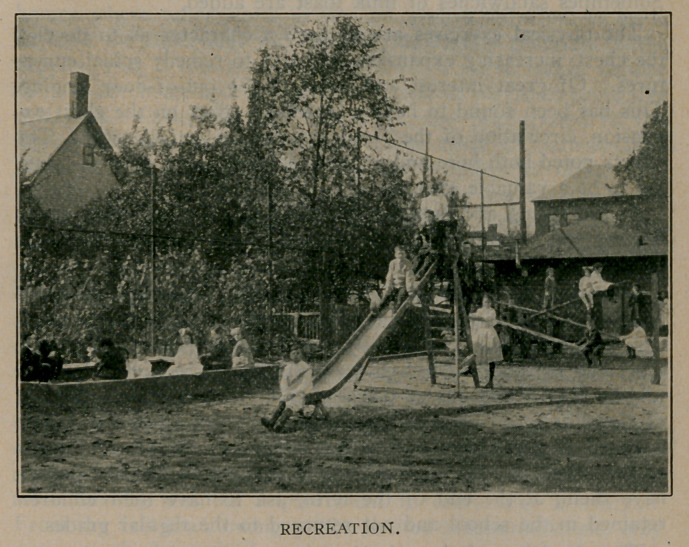# Open Air Schools as a Factor in the Prevention of Disease

**Published:** 1913-08

**Authors:** John Aikman

**Affiliations:** Rochester. N. Y.; Attending Physician at Rochester Open Air School


### Open Air Schools as a Factor in the Prevention of Disease

BY JOHN AIKMAN, M.D.
Rochester, N. Y.
Attending Physician at Rochester Open Air School

[Illustrations through the courtesy of the Rochester Public Health Association.]

IT is quite remarkable that such a wonderful agent as the Open Air School has proved itself to be was never introduced until about eight years ago, and that none were opened in this country until 1908. For years we had known of the tubercle bacilli and of the fact that lowered resistance gave the best chance for this enemy of man to carry out its terrible work. Still we would crowd children from homes where the disease existed into poorly ventilated school rooms, under conditions that made it very difficult to combat the disease to which they were especially susceptible. There were also in these school-rooms, poorly nourished and anaemic children, those with tubercular glands, tonsils, adenoids and many other conditions that made them susceptible to infection. These children were unable to keep up with their grades, would lose several days each term, and were ready victims of every contagious disease that found its way into the school.

In 1904 some of the school workers in Germany thought of the happy plan of teaching these children in the open air. People had awakened to the fact that fresh air, even at a low temperature, was of the greatest need to the growing child. All methods of ventilation had been found to be imperfect and, certainly, none offered a suitable substitute for out-of-doors living. The idea at once became popular and has spread with great rapidity.

The children that have been treated in such schools are not, strictly speaking, hospital cases, and are not ill enough to require treatment at home. They are simply predisposed to disease, and the greatest work of the Open Air School is to increase resistance and thereby avoid serious infection. The parents will not consent to have their children away all the time and many parents, considering, strange to say, the education of the child of more importance than the health, will not consent to removal from school. The Open Air School stands between the school and the hospital, offering a happy solution to the problem. A hospital for the care of such cases would be much more expensive and it would certainly be impossible for a city to treat such cases for months, sometimes a year or two, as is frequently required, before the proper physical standard is reached.

Although much has been said and written in regard to Open Air Schools, it might be well to describe, briefly, a typical school, because it seems that few physicians have had the time to visit the schools and to look into the advantages offered, at least the comparatively few physicians who refer cases to the school would so indicate. The school at Rochester, which was one of the first to be opened, has nearly all the essential features, and will do very well for descriptive purposes. We have, at present, on one of the playgrounds of the city, near No. 14 School, a wooden tent such as is used in the treatment of tuberculosis, which allows free admission of fresh air and is not heated. It simply supplies pro-

AT WORK IN GARDEN.

tection from wind, rain and snow. Near by, in the school building proper, is a large room used for kitchen and dining room. This, of course, is heated, and is used only at meal time. In this building is also a bath room where each week the children are given a thorough bath and inspection by the visiting nurse. All the time possible is spent in the open air and the children go outside the tent for study and exercise when the weather permits. Two teachers and a trained dietitian have charge of the school.

The equipment is inexpensive and the running expenses of the school are quite moderate. The daily food for the children costs only 14 cents per capita. There are at present thirty-five pupils, but as the Board of Education is considering the erection of a larger building with strictly modern equipment at the Cobb's Hill Park, places for many more will soon be provided. With this new school on the outskirts of the city, it is believed that even better results will be obtained.

The object of the school, primarily, is to prevent the development of tuberculosis in predisposed children. With this in view, the cases selected for the school include children from homes where the disease exists, together with cases of anaemia, malnutrition, tubercular glands and underdevelopment. Cardiac, post-operative and cases convalescing from bronchitis, etc., have

DINING ROOM

also been admitted. The most important point in regard to the class taken is that no case that has pulmonary tuberculosis is admitted to the school, even though the disease is incipient. Fortunately there is an Open Air School for these cases at the County Hospital for Tuberculosis (Iola).

Each applicant for admission is examined by Dr. Whipple of the Rochester Public Health Association, and, if the child is in need of such care and no tuberculosis can be detected, he is admitted. However, if tuberculosis develops, the child is immediately transferred to the school for tubercular children at Iola.

Owing to the fact that we have, at present, but one school room, only children between 7 and 14 years of age are received. In regard to the children, I might say here that they so enjoy the school that they are willing to continue through their vacations and, in some cities, have even attended on Saturdays and Sundays. It is easy to imagine that this attitude on the part of the children has much to do with the excellent results obtained.

In winter the children are provided with Eskimo suits, gloves and felt boots. The Eskimo suits are made of woolen blankets so cut and fitted that they will allow free motion and at the same time keep the children warm. The hoods attached to the blanket suits provide protection for the heads.

REST PERIOD.

Following is the routine observed at the school:

| | | |
|---|---|---|
| 8:40- 9:00 | A. M. | —Prepare for breakfast. |
| 9:00- 9:30 | A. M. | —Breakfast. |
| 9:30-10:30 | A. M. | —Lessons. |
| 10:30-10:50 | A. M. | —Gymnastics and games. |
| 10:50-11:00 | A. M. | —Milk. |
| 11:00-11:45 | A. M. | —Lessons. |
| 11:45-12:00 | M. | —Prepare for dinner. |
| 12:00-12:40 | P. M. | —Dinner. |
| 12:40- 2:00 | P. M. | —Rest in reclining chairs. |
| 2:00- 2:10 | P. M. | —Fold blankets and chairs. |
| 2:10- 2:20 | P. M. | —Breathing and rhythm exercises. |
| 2:20- 2:45 | P. M. | —Playground. |
| 2:45- 3:15 | P. M. | —Lessons. |
| 3:15- 3:30 | P. M. | —Prepare for dismissal. |
| 3:30- | P. M. | —Milk and crackers, soup or cocoa. Dismissal. |

The order of exercises is followed, except in cases of cardiac disease, when the physical exertions are limited as indicated by the condition present.

The education of the child is considered of secondary importance to his physical condition, and the studies are made as

RECREATION.

practical and interesting as possible. It is rather surprising to note "the fact that children can do the same amount of work in much less, sometimes in half the time, with increased energy and alertness." Most of them keep up with their regular grades. They are taught practical hygiene and soon develop habits of cleanliness, politeness and good nature owing to the fact that the school is quite like one large family.

The food given at the school has much to do with the good results obtained, and, with what the children receive at home, gives a very nournishing diet. Estimating that the growing child requires 80 calories per kilogram, the boys of this school require 2000 and the girls 1600 calories per day. At the school they receive about 1151 calories of food per capita, so it is seen that over one-half of the total food required is supplied. The food they receive at home more than makes up the balance.

The breakfast at 9 A. M. consists of a cereal with sugar, cream and a glass of milk. At 11 o'clock each child receives a glass of milk or a cup of cocoa. Dinner is served at 12:30 and consists of meat, or a meat substitute, potatoes, one other vegetable, bread, butter, milk and a single dessert or they are given a soup which is high in nutritive value, bread, butter and pudding. The lunch at 3:30 consists of cocoa and crackers or bread and milk. Sometimes sandwiches or milk toast are added.

The physical exercises are of such a character as to develop the chest, increasing expansion, and also to remedy spinal curvatures. Of great interest and value is the out-of-door singing. This has been found to have a beneficial effect on the chest expansion, circulation of the blood, metabolism and digestion. The effects noted both here and in other schools prove open air singing to be a valuable exercise.

In the spring and summer the children have a garden where flowers and vegetables are grown and where the children make nature studies.

The exercises in the school have a marked effect on the mental condition of the child and there is soon shown an increased alertness and attention, and greatly improved personal appearance, especially marked in children from the poorer sections. We are told by the visiting nurses that the children carry home the ideas of cleanliness and hygiene and that many times a marked change is noted in the homes due to this influence. The best friends of the school are the mothers and it is quite the usual thing to have them, at the end of the term, ask to have their children retained in the school and not returned to the regular grades.

The physician of the school makes weekly visits, and every child is examined once a month, records being kept of height, weight, chest expansion, haemoglobin, pulse and temperature. The heart, chest, throat and glands are also examined at the same time. By examining but eight or nine children at a visit it is possible to cover the whole school in a month, give each child sufficient time and not to over-tax the examiner.

By frequent examinations and accurate records it is possible to detect disease at an earlier stage than would be possible in the regular schools. In this way the few cases that develop tuberculosis can be at once removed to the hospital while the disease is incipient. The early detection is, obviously, very beneficial to the child and at the same time removes a source of danger from the school. Any failure to gain weight is at once investigated, and, if nothing is detected on physical examination, inquiries are made at the home as to how the hours outside of school are spent. This, as a rule, accounts for the trouble.

The results obtained show at once the value of this method of treatment.

First, in relation to tuberculosis: As stated above, we try to secure children before the tubercle bacilli have gained a foot hold, in the "pre-tubercular stage." Many cases are obtained from homes where tuberculosis exists and always an effort is made to have the tubercular members of the family removed to the hospital and, if this is not accomplished, a nurse makes regular visits to the home and instructs the family in regard to protective measures. Cases that are run down and predisposed to the disease are also taken, especially those cases of malnutrition, anaemia, round shoulders and the like. Nearly all these cases gain in weight, and during the last term but one has developed the disease. The whole order of the school, with the fresh air, sunlight, food, rest, breathing and other exercises, singing, etc., unite to give very good results. In the principal's report from September, 1912, to February, 1913, we find that forty-one children in all were cared for, eighteen boys and twenty-three girls. All except one, the case which developed tuberculosis, show gains in weight. The greatest gain was 10¼ and the least 1¼ (in seventeen days); the average gain was 3⅝ lbs. Nearly every case shows a gain in chest expansion and improved mental condition. Thirty-seven children show an increase in haemoglobin, four remained stationary and none lost. They all look more healthy and the improvement in every way is marked. From the fact that, owing to the limited accommodations, only cases greatly in need of open air treatment have been received, and that eight out of thirty-five were discharged as cured on February 1st, 1913, to return to their regular schools, it can easily be seen that the right results are obtained.

In regard to other diseases: The class of children admitted are more susceptible to any infection than the average child, and, by staying in the open air, where communication of diseases is more difficult than in the closed school room, and also by increasing the general resistance of the children, epidemics of contagious diseases are much less apt to occur. This is also aided by recording temperatures, keeping the children clean and by exclusion of any suspicious cases.

It has often been noticed that the children in open air schools are quite free from colds which also lessens the likelyhood of development of throat, ear and lung conditions. All cases of enlarged tonsils and adenoids are operated upon before admission, or as soon after admission as possible, and the teeth are examined and treated, thus giving the child a better chance to profit by the open air study.

Other diseased conditions have also been greatly improved, including anaemia, malnutrition, tubercular adenitis and nervous troubles. Cases of cardiac disease have been admitted and, by regulating the physical exercises and giving plenty of rest, good results have been obtained. Any condition in childhood that requires a general upbuilding of the physical condition, will show improvement in the Open Air School.

There is no doubt in the mind of the writer that a large number of the children who have attended the school this year would have become public charges, had they not had advantages here offered. So we may say that, out of the eight children discharged February 1st, practically all were saved to the community.

There are 20,000,000 children attending the public schools of the United States, and it is estimated that 600,000 are in urgent need of open air instruction. At present there are 12 public open air schools in New York City with 526 pupils and forty-one schools in the rest of the United States, in all 1,755, which is very low considering the vast number of children in our cities in need of just such treatment. Such schools are rapidly increasing and are now recognized by all students of public health as being indispensable. Knopf of New York makes the following broad statement. "If we really mean to wipe out tuberculosis from among the coming generation, Open Air Schools and open air instruction should be the rule, indoor schools and indoor instruction the exception."

The schools must have the support of the medical profession, and what is most needed is for the general practitioner and school physician to bear in mind that such institutions exist, and refer children to them; and not only refer, but advise and insist that such a course is necessary.

(880 Monroe Avenue.)

May 1, 1913.

---

ABERHALDEN'S SERO-DIAGNOSIS OF PREGNANCY. Henry Schwartz of St. Louis, *Interstate Med. Jour.*, March, 1913, reviews the literature and explains the technic. In over 300 cases reported, the plus minus error is said to be zero. Positive reactions are found from about the sixth week to 10-15 days after the termination of pregnancy, whether at term or prematurely and irrespective of lactation.

---

MENSTRUATION IN INFANT. Wm. F. Smith of Calexico, Calif., *Southern Calif. Pract.*, March, 1912, reports a case, menstruating on the 7th and 35th days after birth, the first menstruation being accompanied with milk in the breasts.

## Figures and Tables

**Figure f1:**
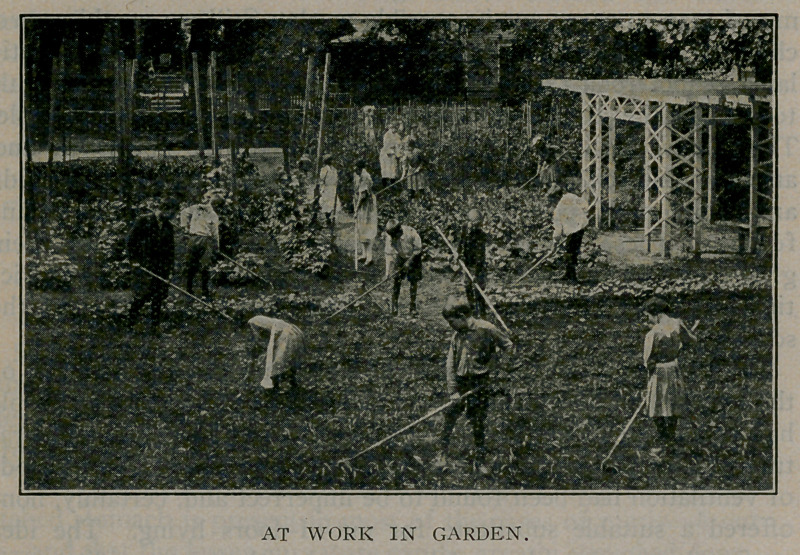


**Figure f2:**
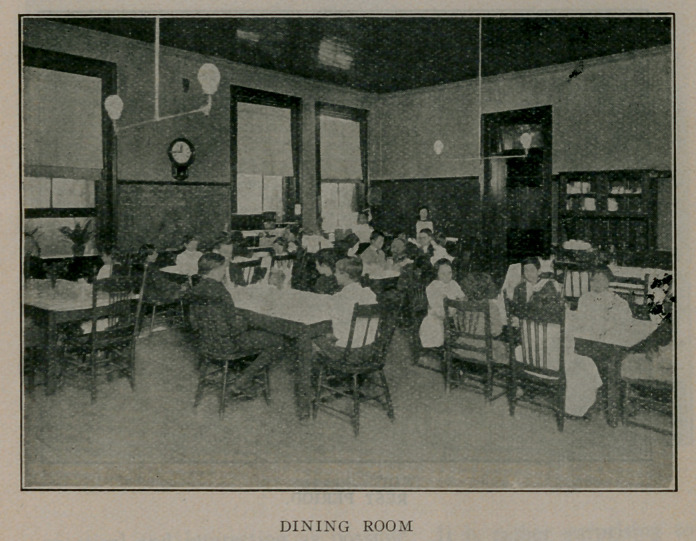


**Figure f3:**
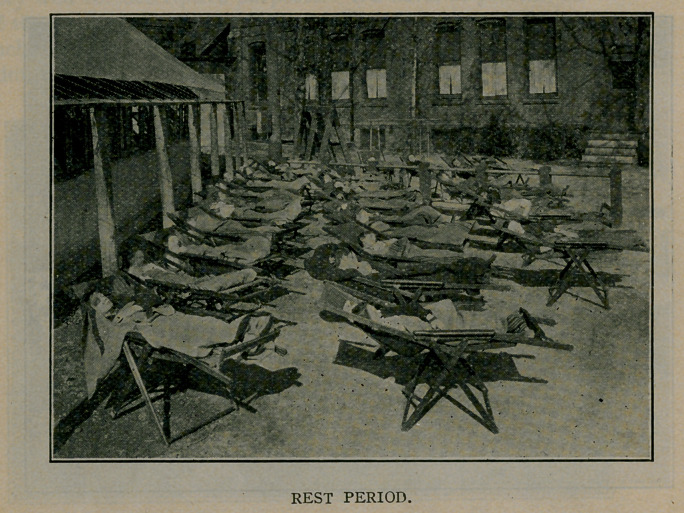


**Figure f4:**